# The Book World of Medicine and Science

**Published:** 1899-07-01

**Authors:** 


### The Book World of Medicine and Science.

Clinical Lectures on Neurasthenia. By Thomas Savill, M.D. (Henry J. Glaisher, Wigmore Street. Pp. 144. 5s. net.)

When medical superintendent of the Paddington Infirmary, Dr. Savill utilised the enormous mass of material lying around him, and to this and to his clinique at the West End Hospital we are indebted for the present volume. The author thinks that many so-called functional diseases are due to anatomical changes, and that these changes could be detected if improved apparatus existed; and he is inclined to believe that chorea and infantile paralysis may be due to a microbe. A similar view of general paralysis was long since taken by some alienist physicians, but we are not aware that researches have as yet established the truth of the idea. Assuredly in the manufacture of toxic substances in the body we have a frequent cause of disease, and this fits in with the clinical facts in some forms of melancholia. Dr. Savill insists on the non-identity of hysterical and functional diseases, and it is easy to see the clinical importance of this distinction. It is often said that the hysterical imitation of disease is under the control of the patient and may at any time be given up, but this is a view we have never been able to take, and we doubt whether an absolutely healthy brain ever did evolve the idea of imitation of disease and carry it out to any great length. Dr. Savill believes that such imitations are due to the involvement of the same structures in some pathological process, and this is a very probable explanation of such conditions. Such are some of the principles enunciated in the first of these lectures, and the remaining five are, so to speak, based on them. In the second lecture the functional group of nervous diseases is shown to be the largest coming before the practitioner, and a more favourable view is expressed of the curableness of nervous diseases, whether functional or not, than the majority of physicians at present hold, but certainly the author's exceptional experience and the cases detailed in the volume warrant his conclusions. We are informed that 90 per cent. of nervous diseases are cases of neurasthenia, and it is carefully pointed out that this is not a new name for hysteria; but that the apparent resemblance in those diseases is to be accounted for by the fact that cases of neurasthenia are often placed among the hysterical. It is the classification that is wrong, not the diseases that are identical; and the distinctions are so explicitly pointed out that no one who reads the book should confuse them again. It is shown that the blood tension is almost invariably low in cases of neurasthenia. Lecture the third treats of the diagnosis of neurasthenia, especially distinguishing it from hypochondriasis, hysteria, and lithæmia; and the connection between dyspepsia and neurasthenia is fully gone into in this lecture. It would seem that, like other nervous diseases, this is on the increase among us; and even after making every allowance for the previous confusion between hysteria and neurasthenia, there would seem to be no resisting the conclusion. The treatment often resolves itself into an attack on the dyspepsia, and in the employment of the least harmful hypnotics; but it can hardly be said that any particularly new line is struck by Dr. Savill. Rest, galvanism, Turkish baths, sea voyages, and in some instances tobacco smoking are recommended in addition to the more purely medicinal treatment. The concluding chapter deals with the relation of neurasthenia to insanity, and yet another attempt is made at a classification of insanity into which a new group, the neurasthenic, is introduced. The book is carefully put together, the statements clear, the arguments easy to follow, and it cannot fail to be a useful guide to the diagnosis and treatment of a prevalent disease.

Remedies for the Needless Injuries to Children Involved in the Present System of School Education. By Clement Dukes, M.D.London, M.R.C.P., J.P. (London: Rivingtons. 1899. Price 1s.)

Dr. Clement Dukes knows so much about boys, having been for a long series of years physician to Rugby School, and his views regarding education and its real aims have about them so strong a tinge of common sense that we are bound to receive with the greatest respect what he says concerning the injurious tendencies of much of the machinery and many of the methods now in use for the training of youth. Dr. Dukes founds all that he says upon the broad principles that education should have for its aims not the gaining of prizes or distinction, or the winning of credit for the school, but the fitting of the boy for the life he has to lead. "School time," he says, "is the principal period in his history where far-seeing provision should be made not only for the development of mind but also for the growth of body and stability of character." This being so, it is sad to see how largely this important period in a child's life is devoted to objects which, instead of helping, only tend to hinder the main object of education. The defects in our educational methods tell with especial hardness upon the younger boys. The development of mind and the development of character are alike dependent upon efficiency of body, and in the same way the pressure on the intellectual side of the brain, as shown in the too early attainment of scholarship in the young, is but too apt to occasion on the moral side instability of character, or to lead to what may be termed "want of backbone." "The less the prodigy or the genius is taught in his early years, the ampler is the promise for his ultimate welfare; yet he is so urged by parents, from conceit or penury, and by teachers as an advertisement of the school, that he is frequently 'used up' physically, and therefore mentally, before his tissues have attained maturity." "The happiest future," Dr. Dukes says, "frequently awaits those children whom the teacher considers dull, and who *will not* be pressed during their early years; for it is they who become vigorous and capable for the battle of life." He maintains that the brain which ripens late is often that which is of most avail in manhood, and he makes the very interesting remark that "it is a question whether the children of long-lived parents do not always take a longer time in coming to maturity." Contrary to what is thought by many parents, who hearing more of cricket than of lessons are apt to grumble that the time of education for which they pay so dearly is mostly spent in games, Dr. Dukes maintains that school children are often very seriously overworked, and there can be very little doubt that he is right in saying that an early scholarship is often positively hurtful to a child, and that the recipient of these distinctions should on entering school be placed at least two forms below the standard of work which he has reached under the pressure to which he has been subjected. In fact education should be a levelling up of all the faculties rather than a forcing of the one which happens to be most in evidence. "It is almost inconceivable," says Dr. Dukes, "that it never occurs to parents or teacher, where a child is largely brains without physique, that the chief aim in its education should be the development of the body at the only stage when it is plastic, with the concurrent resting of the brain in aid of this effort. . . . I continually see children whose mental faculties are abnormally acute, but housed in the puniest and poorest frame. Such a partially developed child, however, is constantly passed on in the one direction to the increasing atrophy of his body, and the ultimate degeneration of the whole being." This is a pamphlet full of common sense and well worthy of perusal by those who are engaged in the education of youth.

